# Electroencephalographic Findings, Antiepileptic Drugs and Risk Factors of 433 Individuals Referred to a Tertiary Care Hospital in Ethiopia

**DOI:** 10.4314/ejhs.v32i5.5

**Published:** 2022-09

**Authors:** Biniyam A Ayele, Hanna Demissie Belay, Dereje Melka Oda, Yohannes D Gelan, Hanna Assefa Negash, Selam Kifelew, Meron Awraris Gebrewold, Teklil Hagos, Yared Z Zewde, Yared Mamushet Yifru, Fikru Tsehayneh, Amanuel Amare, Ayalew Moges, Seid Ali Gugssa, Abenet Tafesse Mengesha

**Affiliations:** 1 Department of Neurology, College of Health Science, Addis Ababa University; 2 Department of Paediatrics and Child Health, College of Health Science, Addis Ababa University

**Keywords:** Electroencephalogram, epilepsy, epileptiform discharges, risk factors, Ethiopia

## Abstract

**Background:**

Little is known about the characteristics of electroencephalogram (EEG) findings in epileptic patients in Ethiopia. The objective of this study was to characterize the EEG patterns, indications, antiepileptic drugs (AEDs), and epilepsy risk factors.

**Methods:**

A retrospective observational review of EEG test records of 433 patients referred to our electrophysiology unit between July 01, 2020 and December 31, 2021.

**Results:**

The age distribution in the study participants was right skewed unipolar age distribution for both sexes and the mean age of 33.8 (SD=15.7) years. Male accounted for 51.7%. Generalized tonic clonic seizure was the most common seizure type. The commonest indication for EEG was abnormal body movement with loss of consciousness (35.2%). Abnormal EEG findings were observed in 55.2%; more than half of them were Interictal epileptiform discharges, followed by focal/or generalized slowing. Phenobarbitone was the commonest AEDs. A quarter (20.1%) of the patients were getting a combination of two AEDs and 5.2% were on 3 different AEDs. Individuals taking the older AEDs and those on 2 or more AEDs tended to have abnormal EEG findings. A cerebrovascular disorder (27.4%) is the prevalent risk factor identified followed by brain tumor, HIV infection, and traumatic head injury respectively.

**Conclusions:**

High burden of abnormal EEG findings among epileptic patients referred to our unit. The proportion of abnormal EEG patterns was higher in patients taking older generation AEDs and in those on 2 or more AEDs. Stroke, brain tumor, HIV infection and traumatic head injury were the commonest identified epilepsy risk factors.

## Introduction

Epilepsy is one of the most common neurological diseases, affecting nearly 50 million people of all ages around the world. Majority (80%) of epileptic patients lives in low- and middle-income countries; most of them do not have access to advanced diagnostics and treatment (1). An estimated 25% of epilepsy cases are preventable. The major modifiable risk factors for epilepsy are: perinatal insults, central nervous system infections, traumatic brain injury and stroke (1). Electroencephalogram (EEG) is a recording of cortical electrical activity by using small scalp electrodes. Thus the EEG is a spatiotemporal average of synchronous postsynaptic potentials arising in radially oriented pyramidal cells in cortical gyri over the cerebral convexity (2). EEG is the best neuro-diagnostic method for detecting cortically originating epileptic activity (2–5). Because of this, EEG remains the most important investigative modality in the diagnostic evaluation of individuals with epilepsy. Epilepsy diagnosis is clinical, but EEG helps to establish the diagnosis, classify seizure types, and prognosticate the patient (2,3,6–11). One of the challenges of epilepsy care in resource limited areas is limited access to EEG especially in sub Saharan Africa (SSA) there is a lack of access to EEG service (6,7). Furthermore, in SSA access to a quality EEG service is very limited; due to limited trained EEG technicians and neurologists (6,7).

In Ethiopia, little is known about the characteristics of electroencephalogram findings in epileptic patients. According to a review done by Deresse et al. 2012 (12), out of 251 reviewed EEG recordings, 59.4% showed abnormal EEG findings; the majority of these abnormalities (61.1%) were epileptiform discharges, the remainder (38.9%) being nonspecific patterns (12). EEG provides information that primarily concerns disturbances of function rather than structure (3). According to Biniyam et al. (13), 52.5% of patients with Parkinson's disease showed diffuse slowing on routine resting state EEG recordings.

The objective of the present study was to characterize EEG pattern, indications for the EEG referral, antiepileptic drugs, and epilepsy risk factors in individuals referred for EEG recordings. All the EEG recordings were for 30 minutes and standard activation procedures such as hyperventilation and photic stimulation were used in all patients with the following exceptions, no hyperventilation for those ages above 60 years and no photic stimulation for infants below age 6 months.

## Methods and Materials

**Study setting, design and period**: The study was conducted at electrophysiology unit at Tikur Anbessa Specialized Hospital (TASH), a tertiary level university hospital located in Addis Ababa, Ethiopia. The electrophysiology unit is equipped with two NIHON KOHDEN 32 channel EEG machine and two nerve conduction study and electromyography machines. EEG is done on a daily basis on working days and patients are sent from the neurology outpatient clinics, the ER, medical/neurology in-patient services, other medical and surgical units and from other hospitals through a referral as well. This is a facility based retrospective observational review of 433 EEG test records of all consecutive patients who were referred to the electrophysiology unit between July 01, 2020 and December 31, 2021. In this study only few children were included, this is because, and the electrophysiology unit is primarily serving adult neurology patients. Hence, most of the included patients were adults.

**EEG recordings and interpretation**: All 433 EEG were recorded in a resting state and conducted by 5 trained EEG technicians working at the electrophysiology unit using a 10–20 international system. The EEG technicians instructed the patients to sit upright in a quiet and dimly shielded room with eyes closed to attain a state of relaxed wakefulness. The patients were instructed not to fidget, talk, or move to avoid movement artifacts during the recording process. Each EEG recording lasted 30 minutes. The standard activation procedures such as hyperventilation and photic stimulation were used in all patients with the following exceptions, no hyperventilation for those ages above 60 years and no photic stimulation for infants below age 6 months. Since the electrophysiology unit has no sleep lab, no formal sleep analysis was included in the present review. However, very few patients had evidence of sleep architecture and those were reported under benign EEG variants. All the recorded EEG tracings were interpreted and reported by certified neurologists (BAA, HD, DM, YD, FT, YZ, MA, SA, and AA). Of these neurologists, three of them (BAA, MA, & YD) have attended past International Federation of Clinical Neurophysiology (IFCN) training fellowships.

**Ethical approval of the study protocol**: The study received ethical approval from Addis Ababa University, College of Health Science Ethical Clearance Committee (Protocol number· 003/20/Neuro).

**Statistical analysis**: The demographic characteristics, seizure classifications, antiepileptic drugs, epilepsy risk factors, and EEG findings were described using means, median, frequency, percentile, and standard deviation and the results were presented using summary tables and graphs.

## Results

**Figure 1:**
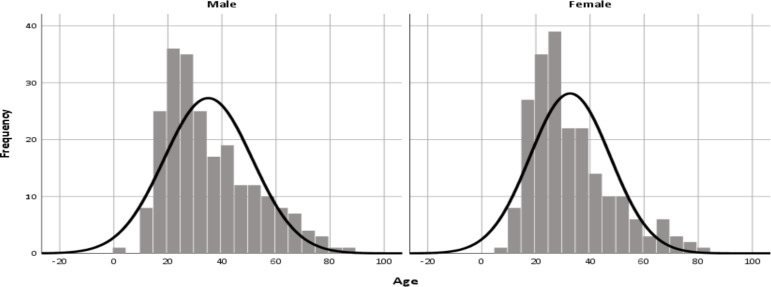
Histogram showing right skewed unipolar age distribution of the study participants

**Baseline characteristics and clinical profile of the study participants**: In the present review, a total of 433 EEGs of individuals referred to the unit were analyzed. Male accounted for 51.7% (n= 224). The age distribution in the study participants was right skewed unipolar for both sexes (Figure 1). The mean age was 33.8 (SD=15.7) years. Majority of our patients (90.5%) were right-handed. Most of the EEGs (86.2%) were referred from neurology referral clinics. Small proportions of the patients were referred from the emergency room and intensive care unit. A quarter of the individuals had a previous EEG record (Table 1). Generalized tonic clonic seizure was the most common seizure type (45.5%) followed by focal onset seizure (21.7%). Mixed seizure type was seen in 6% of our epileptic patients. In the present review, 5.5% reported comorbid hypertension, Psychiatric disorders (3.2%), and 3% had cardiac diseases (Table 1).

**Table 1:** Baseline characteristics of study participants (n=433)

Variables		Values
Age in years (mean, SD)		33.8 (15.7) years
Gender (n, %)	Male	224 (51.7)
	Female	209 (48.3)
Handedness (n, %)	Right	392 (90.5)
	Left	26 (6)
	Not reported	15 (3.5)
EEG referring units (n, %)	Neurology referral clinic	355 (82)
	Medical/neurology ward	18 (4.2)
	Emergency department	7 (1.6)
	Other departments	11 (2.5)
	Outside TASH	2 (0.5)
	Referring unit not mentioned	36 (8.3)
Previous EEG recordings (n, %)	Yes	98 (22.6)
	No	264 (61)
	Unknown	71 (16.4)
Seizure classification* (n, %) (N=417)	Generalized tonic clonic (GTC)	197 (45.5)
	Generalized atonic	2 (0.5)
	Absence	11 (2.5)
	Myoclonic	7 (1.6)
	Focal	67 (15.5)
	Focal to bilateral tonic clonic	27 (6.2)
	Mixed seizure type	26 (6)
	Seizure type not specified	69 (15.9)
Comorbid diseases (n, %)		
	Diabetes mellitus	11 (2.5)
	Hypertension	24 (5.5)
	Cardiac diseases	13 (3.0)
	Psychiatric illness	14 (3.2)
	Migraine headache	10 (2.3)
	Others	37 (8.5)
	Comorbidity not reported	324 (74.8)

* In all the variables, the denominator is (N=433), unless specified otherwise. TASH: Tikur Anbessa Specialized Hospital; EEG: Electroencephalography; SD: Standard deviation

**Figure 2:**
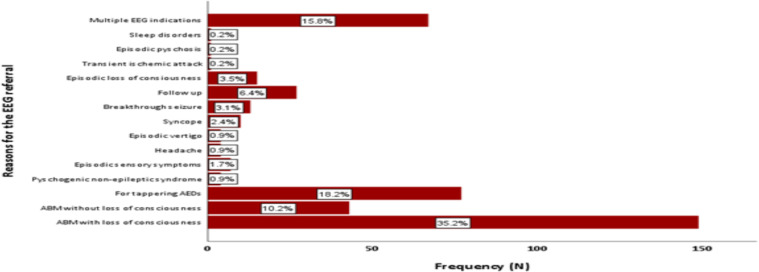
Bar graph showing distribution of reasons for EEG referral in the study participants (¶TASH: Tikur Anbessa Specialized Hospital; EEG: Electroencephalography; ABM: Abnormal body movement; AEDs: Antiepileptic drugs)

**Reasons for EEG referrals, antiepileptic drugs, and epilepsy risk factors**: The commonest indication for EEG referral in the present review was for diagnostic purpose in patients presenting with abnormal body movement with loss of consciousness (35.2%) followed by EEG request for tapering antiepileptic drugs (AEDs) (18.2%). Around 15.8 % of patients referred have multiple indications for EEG. Furthermore, psychogenic nonepileptic syndromes, episodic loss of consciousness, breakthrough seizure and syncope among others were listed as a reason for EEG referral (Figure 2).

**Figure 3:**
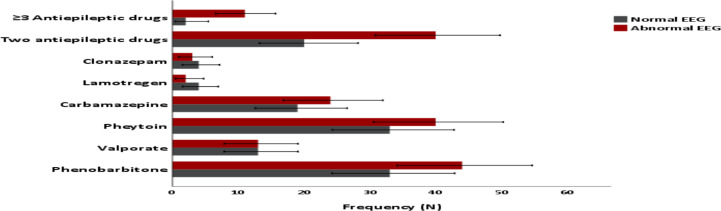
Bar graph showing distribution of AEDs used to control seizure in the study participants. (¶AEDs: Antiepileptic drugs; EEG: Electroencephalography)

In this review, the most commonly used AED was phenobarbitone followed by phenytoin, carbamazepine and valproate. Minority of patients were also on lamotrigine and clonazepam. Of those on AEDs, 20.1% of the patients were getting a combination of two AEDs and 5.2% were on 3 different AEDs (Figure 3). Furthermore, epileptic patients on phenobarbitone, phenytoin, carbamazepine, and those on 2 or more antiepileptic medications were found to have higher proportion of abnormal EEG findings (Figure 3). The proportion of normal EEG recordings was higher in epileptic patients taking clonazepam and lamotrigine. Equal proportion of normal and abnormal EEG was observed in those epileptic patients on valproate (Graph 3).

**Figure 4:**
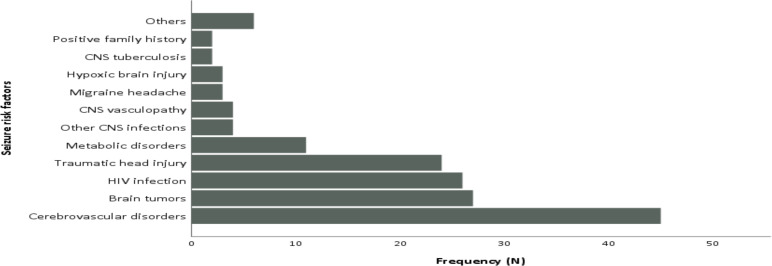
Bar graph showing distribution of seizure risk factors in the study participants. (¶CNS: Central nervous system; HIV: Human immunodeficiency virus)

In the present review, in 37.8% one or more epilepsy risk factors have been observed. A cerebrovascular disorder (27.4%) is the prevalent risk factor followed by brain tumor, HIV infection, and traumatic head injury (Figure 4). Positive family history, CNS tuberculosis, CNS vasculopathies, and others accounted for a small percentage (Figure 4).

**Electroencephalograph findings in the study participants**: The majority of the study participants (n=369, 85.2%) had an alpha background rhythm. Almost all of the participants 419 (96.8%) were awake and communicative during the EEG recordings, where only 6(1.4%) were comatose. Among the 433 EEG recordings 55.2% (n=239) of them harbors abnormal EEG findings (Table 2). Among the abnormal EEG recordings, more than half of them were epileptiform discharges. Most of these epileptiform discharges, 120(91%) were Interictal epileptiform discharges and 9% showed ictal discharges (Table 2). More than half of the Interictal epileptiform discharges, 57.5% accounted for generalized epileptiform discharge, whereas only 7.2% these EEG recordings were typical for 3Hz generalized epileptiform discharges. In very few patients, 5 (4.2%) period lateralized epileptiform discharges (PLEDs) were noted (Table 2). The rest 107 (44.8%) of the abnormal EEG recordings were slowing's, of which focal slowing was noted in almost half of these, 57(53%) of the recordings. Generalized non rhythmic type of slowing is the common type of general slowing noted in 35 (70%) of the generalized slowing. Among the generalized rhythmic slowing, Temporal Rhythmic Intermittent Delta activities were the most common rhythmic slowing being noted in 14 patients (93.3% of rhythmic slowing) (Table 2).

**Table 2:** Electroencephalograph (EEG) findings in the study participants (n=433)

Variables		Frequency (%)
Mental status during EEG recording	Awake and communicative	419 (96.8)
	Lethargic	8 (1.8)
	Comatose	6 (1.4)
EEG background rhythm	Beta (>13 Hz)	2 (0.5)
	Alpha (8 – 13 Hz)	369 (85.2)
	Theta (4 – 7 Hz)	52 (12.0)
	Delta (0.5 – 3 Hz)	7 (1.6)
	Mixed background rhythm	3 (0.7)
Hemispheric lateralization* (N= 217/433)	Bilateral hemisphere	90 (41.5)
	Right hemisphere	63 (29.0)
	Left hemisphere	64 (29.4)
Lobar involvement* (N=158/433)	Temporal	74 (46.8)
	Frontal	26 (16.5)
	Parietal	4 (2.5)
	Parasagittal region	1 (0.6)
	Multilobar involvement	53 (33.5)
Normal & benign EEG variants		194 (44.8)
Epileptiform discharges		132 (55.2)
Interictal discharges (n=120/132)		120 (91)
Generalized epileptiform discharges		69 (57.5)
3 HZ generalized epileptiform discharges(n=5/69)		5 (7.2)
Focal epileptiform discharges		46 (38.3)
Periodic lateralized epileptiform discharges (PLEDs)		5 (4.16)
Ictal discharges (focal + generalized) (n= 12/132)		12 (9.1)
EEG Slowing		107 (44.8)
Generalized slowing (N=50/107)		50 (46.7)
Generalized non rhythmic slowing (episodic + diffuse) (n=50)		35 (70)
Temporal rhythmic intermittent Delta activities (TRIDA) (n=15)		14 (93.3)
Frontal rhythmic intermittent Delta activities (FRIDA) (n=15)		1 (6.7)
Focal slowing (n= 57/107)		57 (53.2%)

## Discussion

The present study shows a high burden of abnormal EEG findings in individuals referred to our electrodiagnostic unit. The age distribution was a unipolar with right skewed age distribution for both males and females; with mean age in the third decades. This is in congruent with similar reports from Ethiopia (14,15). However, it contradicts the western studies which showed a bimodal age distribution; epilepsy occurring frequently among the younger and the oldest (16–18). This is because, in high income countries (HIC), epilepsy is common among younger children and older adults (i.e bimodal pattern); likely due to high prevalence of perinatal disorders and strokes respectively. Contrary to this, in the present review the mean age was in the third decades, pointing to traumatic brain injury and neuroinfectious diseases as possible etiology of epilepsy(8,12,18–22). Furthermore, these findings will guide future interventions in Ethiopia to reduce the burden of epilepsy in the young and productive segment of the population.

In this review, generalized tonic clonic type of seizure is the most prevalent followed by focal seizure. This is consistent with previous reports (12,15,19,21). Due to resource constraints in LMIC countries, EEG is often done for patients whose EEG is likely to contribute significantly to the patient's care since prioritization may be required. After careful reviewing multiple guidelines and considering the limited resources in Zambia, Bierbek et al suggested to do EEG for selected patients including all unconscious patients suspected of non-convulsive status epilepticus or subclinical seizures and epileptic patients who failed to respond to standard treatments and/or seizures and progressive neurologic problems(23). The practice is more or less similar in Ethiopia. In the present review, two third (63.6%) of EEG were prescribed either for patients who have abnormal body movements or are trying to taper medications off. This is probably due to stringent patient selection for EEG recording due to limited resources in Ethiopia.

EEG is the most specific method to define the epileptogenic cortex; rarely, epileptiform discharges are recorded in healthy, particularly young individuals (24). In the present study, abnormal EEG findings were observed in more than half of the study participants. Of this, the majority of them were epileptiform discharges, while the rest were in the form of slowing, periodic discharges, or rhythmic discharges. This is in congruent with a review reported from Ethiopia by Birre et al (12), where out of 251 EEG, 59.4% harbors EEG abnormalities. Such a high burden of abnormal EEG patterns in epileptic patients in Ethiopia could indicate lack of proper epilepsy treatment, poor AEDs adherence and significant burden of structural abnormalities as an underlying culprit. However, much lower prevalence of EEG abnormalities (18%) was reported from a community-based study in rural Ethiopia (15). The observed difference could be due to the fact that epileptic majority of patients followed at a tertiary care hospital tends to have more severe or poorly controlled epilepsy resulting in artificial inflation of the abnormal EEG findings.

In the present survey, the older generation AEDs such as phenobarbitone and phenytoin were the commonly prescribed medications in epilepsy treatment. Meanwhile, only a very few proportion of epileptic patients were on the newer AEDs. Even though older and newer drugs are likely to have similar effects in terms of seizure control, the newer generation medications tend to have lesser adverse effects, which will improve patients' adherence to their anti-seizure drugs (25). Furthermore, epileptic patients who are taking two or more AEDs were more likely to have abnormal EEG findings. This indicates the likely refractory nature of underlying epilepsy in individuals taking 2 or more medications. In this survey, stroke, HIV infection and traumatic head injury were the commonest identified epilepsy risk factors. This is consistent with similar reports from the region (19,26–30). Limitations of the present study include: being a retrospective review, lack of healthy control group, and lack of quantitative assessment of the EEG results.

The present review shows a high burden of abnormal EEG findings among epileptic patients referred to our unit. The proportion of abnormal EEG patterns was higher in patients taking older generation AEDs and in those on 2 or more antiepileptic drugs. Stroke, HIV infection and traumatic head injury were the commonest identified epilepsy risk factors. We recommend conducting future controlled prospective study to consolidate our findings.
